# Nanostructured Silver Found in Ancient Dacian Bracelets from Cehei Hoard—Salaj, Romania

**DOI:** 10.3390/nano15221740

**Published:** 2025-11-19

**Authors:** Ioan Petean, Emanoil Pripon, Horea Pop, Codruta Sarosi, Gertrud Alexandra Paltinean, Simona Elena Avram, Nicoleta Ignat, Lucian Barbu Tudoran, Gheorghe Borodi

**Affiliations:** 1 Faculty of Chemistry and Chemical Engineering, Babes-Bolyai University, 11 Arany Janos Street, 400028 Cluj-Napoca, Romania; nicoleta.cotolan@ubbcluj.ro; 2 Zalau County Museum of History and Art, 9 Unirii Street, 450042 Zalau, Romania; emanoilpripon@gmail.com (E.P.); horeapopd@yahoo.com (H.P.); 3 Department of Polymer Composites, Institute of Chemistry Raluca Ripan, Babes Bolyai University, 30 Fantanele Street, 400294 Cluj-Napoca, Romania; gertrud.paltinean@ubbcluj.ro; 4 Faculty of Materials and Environmental Engineering, Technical University of Cluj-Napoca, 103–105 Muncii Boulevard, 400641 Cluj-Napoca, Romania; simona.avram@imadd.utcluj.ro; 5 Faculty of Biology and Geology, Babes-Bolyai University, 44 Gheorghe Bilaşcu Street, 400015 Cluj-Napoca, Romania; lucian.barbu@ubbcluj.ro; 6 National Institute for Research and Development of Isotopic and Molecular Technologies, 65–103 Donath Street, 400293 Cluj-Napoca, Romania; borodi@itim-cj.ro

**Keywords:** nanostructured silver, Dacian bracelets, Vickers microhardness

## Abstract

Nanomaterials are usually associated with modern technologies and advanced processing methods. Three silver Dacian bracelets within Cehei hoard (Salaj County, Romania) are tougher than they should be according to the apparently higher silver content. The microstructural investigation reveals that all three bracelets have silver content of about 90 wt.%. The metallographic inspection of a bracelet sample reveals a very refined microstructure of α grain while fewer eutectic grains are almost undetectable, indicating intensive plastic deformation. XRD patterns of the bracelets reveal relevant peaks for silver (without copper) having a much-broadened aspect indicating nanostructural level. The nano-grains were evidenced at high magnification of SEM imaging: 55 nm for bracelet 1, 95 nm for bracelet 2 and 75 nm for bracelet 3. Elemental maps reveal that the nanograins are basically formed by α phase; the finest eutectic traces are situated and uniformly dispersed within α phase, appearing as small red spots. Vickers µHV10 micro indentation was calibrated on a pure silver 999.9 ‰ in annealed state, resulting in 37 HV10. The nanostructured bracelets have about 56 µHV10 for bracelet 1; 50 µHV10 for bracelet 2 and 52 µHV10 for bracelet 3. Dyrrachium drachmas have Vickers microhardness of about 37 µHV10. The obtained results confirm the historian’s supposition that Dyrrachium drachmas could be the source for silver but also clearly indicate that the final steps of bracelets manufacturing were effectuated by cold deformation with intensive cold hardening. It results that cold deformation of the bracelets rods induces a nanostructural state that significantly increases their microhardness instead of their higher silver title.

## 1. Introduction

Archeological science needs help from the other scientific fields for a proper understanding of the artifacts: how were they made and from which materials? Metallic artifacts’ shape and microstructure strongly depend on the manufacturing technological procedures and require specific investigations such as X-ray diffraction (XRD), Scanning Electron Microscopy (SEM) coupled with Energy Dispersive Spectroscopy (EDS), X-ray fluorescence (XRF) and metallographic microscopy when it is suitable [1,2].

It is well known that the intensive cold working of the metals and alloys induces the cold hardening effect which makes the alloys tenacious and steely because of the intensive refining of the grain structure such as in, for example, the cold rolling [3] or cryomilling process [4]. It has certain benefits in tailoring the alloys microstructure, but the excessive cold hardening causes the inter-granular failure and breaking of the worked metallic parts [5,6]. An increased tenacity of the metallic parts requires proper annealing between the cold working sessions in a similar manner to the Japanese samurai swords [7] to allow internal restructuring of the grains [8,9].

The search within international journals databases did not retrieve any results regarding the metallic artifacts’ nanostructure resuming the investigated aspects to their microstructure, a fact understandable if considering the relatively poor metallurgical processes in antiquity compared with the key parameters control nowadays. But the search returns many new results regarding the nanostructured system used for the restoring of ancient artifacts like nanostructured metal oxides used for conservation of cultural heritage [10] and the effect of organo-silicon on the nanostructure of waterlogged archeological oak [11]. Some of the ancient artifact production techniques might cause the grains size after refinement to drop below 99 nm, making them nanostructural by chance, not by intended purpose. For example, Dacian civilization has less written archeological sources but certain references to their culture and practice are found in Roman and Greek sources. There are also a lot of metallic archeological findings (including gold and silver beside bronze) including metallurgical workshops [12,13]. It is very interesting to look for a Dacian hoard discovery.

Discovered on 25 April 1986, in Cehei (the town of Șimleu Silvaniei), the hoard consists of a brooch, three bracelets and a chain, all made of silver, as well as 552 Dyrrhachium drachmas, of which 445 are original and contain no less than 26 associations of names of the issuing magistrates and 7 are imitations [14]. The hoard was discovered in the area of the Dacian settlement of Șimleu Silvaniei-Observator, located on an important trade route: the salt road, which entered Sălaj county from the southeast, passed through the Meseș Gates and headed towards the Crasna Valley through Sărmășag or along the Barcăului Valley towards Marca, reaching Șimleu Silvaniei along the way [15].

The Cehei hoard is part of a series of numerous monetary hoards buried in the Second Iron Age, La Tène D1, sometime between the end of the 2nd century BC and the first two decades of the 1st century BC [15]. Since its publication, it has been hypothesized that the good quality silver in the five objects of adornment (jewelry), valued at a fineness of 800/1000, came from the coins that make up the hoard. At that time, the idea was that a good part of the Dacian ornaments came from the coins that entered Dacia after the middle of the 2nd century BC, namely tetradrachms of Macedonia I, drachms of Dyrrhachium and Apollonia, tetradrachms of Thasos and Roman Republican denarii [14].

The authors assumed that to make the five ornaments, the coins were first melted and then transformed into silver bars during “a quasi-mandatory intermediate stage” for the transformation of coins into ornaments. Taking into account a 10% loss as a result of melting the coins and transforming them into bars, the authors concluded that the raw material for making the five ornaments in the hoard was the equivalent of 120 drachmas of Dyrrhachium [14].

The actual evaluation of the hoard components reveals an increased tenacity of the bracelets, compared to the other jewelry from the hoard (e.g., necklace and fibulae) having a strong steely behavior unspecific to silver. Thus, simply melting the Dyrrhachium drachma and subsequently casting into bars is an insufficient assumption to fully characterize the bracelets. Such toughness would require alloying with hardening elements or, as previously mentioned, the cold working induces an advanced refining of the microstructure, improving its tenacity. Therefore, assuming the typical protocol for investigation of nanostructured materials, the current investigation aims to reveal the precise composition of the bracelets and to figure out the ancient manufacturing technology. The intensive cold working of the bracelets might turn their material into a nanostructured one, a very possible fact which could be revealed by advanced investigations like XRD associated with SEM and EDS.

## 2. Materials and Methods

### 2.1. Samples Description and Preparation

**Figure 1:**
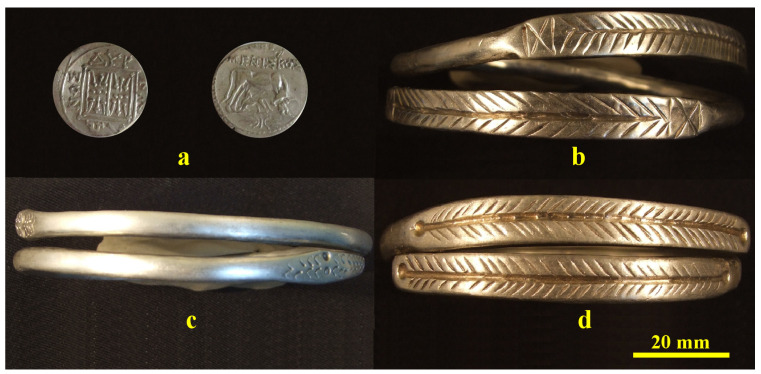
The investigated pieces from Cehei Hoard: (**a**) Dyrrhachium drachma, (**b**) bracelet 1, (**c**) bracelet 2 and (**d**) bracelet 3.

The investigated bracelets belong to the Cehei Hoard, Figure 1, in custody of Zalau Museum of History and Art, Romania. Their shape is typical for the Dacian culture, the terminations being decorated with specific motifs resembling snake heads (bracelet 1) and fir branches in bracelets 2 and 3, Figure 1c,d. These are simple bracelets without spirals, allowing their easy investigation through the nondestructive methods. All hoard pieces are perfectly cleaned and preserved according to the standard of practice for being able to be presented in exhibitions; therefore, they are suitable for direct investigations without any other preparations.

A very small splinter about 1 mm length was gently sampled from the inside part of each bracelet for metallographic investigation. The sampling procedure was effectuated with care to not affect the artifact state. The collected splinters were embedded in Bakelite and subsequently grinded with different granulation sandpapers: 1000, 2000, 4000 and 8000. The final polishing step was performed on a felt disc impregnated with colloidal alumina. The metallographic samples were etched using a swabbing method with a reagent based on ferric chloride and sodium thiosulfate [16].

Since the hypothesis made by the authors who published the hoard discovery mentioned that Dyrrhachium drachmas were used as raw material for bracelets, we selected a representative coin which was investigated for comparison. Their metallographic aspect was revealed on its side through a minimally invasive procedure by vertical embedding in Bakelite, and care was taken to ensure minimal loss during grinding and polishing procedures.

### 2.2. Investigation Methods

The alloy phases and oxide traces from the artifact natural patina were evidenced by the X-ray diffraction (XRD) which was performed on a Bruker D8 Advance (Bruker Company, Karlsruhe, Germany) using Cu kα radiation (1.540562 Å). The patterns were registered at a speed of 1 degree/minute. Peaks assignment was performed with Match 1.0 software equipped with a PDF 2.0 database (Crystal impact Company, Bonn, Germany).

Metallographic microscopy was effectuated on a specific optical microscope IOR MC8 (IOR company, Bucharest, Romania) equipped with a digital capture of the images Sony 14MPX (Sony company, Minato, Japan).

Scanning Electron Microscopy (SEM) coupled with Energy Dispersive Spectroscopy (EDS) was used for the investigation of the morphological aspects correlated with the elemental distribution. Therefore, the morphological aspects were observed on the Secondary electron images (SEI), and the elemental distribution map was displayed on the Backscattered Electron Image (BSE) taken on the same area as SEI image. All these operations were performed with a Hitachi SU8230 microscope (Hitachi Company, Tokyo, Japan) operated in high vacuum mode at an acceleration voltage of 30 kV. Elemental analysis was effectuated with EDS detection module X-Mas 1160 EDX (Oxford Instruments, Oxford, UK) installed on the SEM device.

Transmission Electron Microscopy (TEM) was performed with Hitachi TEM series HD-2700 (Hitachi Company, Tokyo, Japan) on the collodion replicas taken from the samples surface. The images were further analyzed with specific software Image J version 1.53t (National Institute of Health, Bethesda, Rockville, MD, USA).

Microhardness was effectuated with a Duramin40 AC3 device (Struers Company, Ballerup, Denmark) operated with Vickers indenter µHV10 type at a dwell time of 10 s and a load of 10 g. There were at least three indentations taken on each sample (in the less visible side of the bracelets), and the mean value was calculated along with the standard deviation. The mean values were statistically analyzed using Anova method followed by Tukey post hoc test at a relevance level of 0.05. The graphical plot and statistical analysis were effectuated with Origin 9b software (Microcal Co., Amherst, MA, USA).

## 3. Results

### 3.1. Alloy Phases and Their Microstructural Constituents

**Figure 2:**
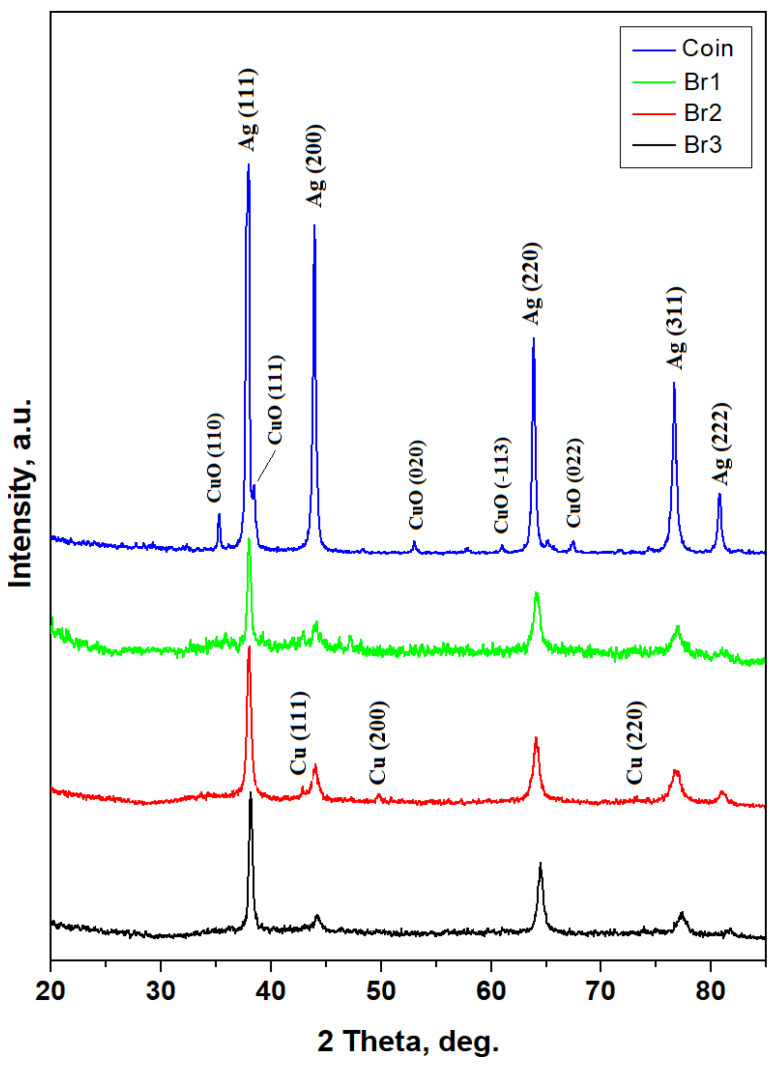
XRD patterns obtained for the investigated pieces from Cehei Hoard. The following cards from the PDF database were used for the peaks assignment: Ag PDF 65-2871; Cu PDF 89-2838 and CuO PDF 89-2529.

The alloys phases (e.g., pure metals or intermetallic compounds) can be detected by XRD with high accuracy, giving advanced qualitative characterization. Thus, Dyrrhachium drachma has a diffraction pattern featuring very well-developed peaks with strong intensities and narrow shape, Figure 2. It corresponds to a well-annealed metallic sample. The drachma composition is dominated by Ag while the smaller amount of copper appears to be oxidized as CuO (crystallized as Tenorite), a fact in good agreement with the antique look of the coin. It makes it difficult to establish a semi-quantitative of the Ag/Cu ratio within.

On the other hand, the XRD patterns resulting from bracelets have a completely changed shape with less intense peaks and broadened width. This aspect clearly indicates advanced refinement of the microstructure most likely induced by the cold working, as shown in Figure 2.

XRD peaks of all three bracelets are dominated by the Ag peaks followed by significantly smaller Cu peaks. The oxide amount on their surface is below diffractometer detection limit and therefore the specific peaks are not observed. It allows applying the Relative Intensity Ratio (RIR) method for establishing the silver amount [17,18]. Briefly, there are two phases which generated diffraction peaks, and they are co-related as follows:

(1) $$\frac{Ia}{Ib}=\left(\frac{xa}{xb}\right)\cdot \left(\frac{fa}{fb}\right).$$

where Ia is the most representative intensity of phase a, and Ib is the same for phase b; xa and xb are the mass percentage concentrations for the two phases and fa and fb are their Corundum factors. Finally, it results that bracelets have the silver amount of about 90–95% regarding copper.

The full width at half height (FWHM) of the diffraction peaks allows calculation of the crystallite size. Scherrer formula is generally used for the mineral samples and for crystalline matter which was not subjected to the intense mechanical stress [19,20]; it is described by Equation (2):

(2) $$D=\frac{k\lambda }{B\cos\theta }.$$

where D represents the crystallite size, k is the Scherrer constant typical 0.9 for metals and alloys, B is FWHM and θ is the Bragg angle.

When the alloy amorphization is induced by mechanical stress like in the mechanical alloying, the remnant strains accumulated into the crystal lattice superimpose a widening effect of the diffraction peaks beside the crystallite refinement. In consequence, the strain component must be subtracted from the crystallite size calculation. Williamson–Hall is one of the effective methods of fulfilling this requirement [21,22]. This method assumes that the FWHM is induced by crystallites size refinement B_size_ as well as due to the remnant strains B_strain_ at the crystal lattice level described as

(3) $$B_{total}=B_{size}+B_{strain},$$

which further becomes

(4) $$B_{total}=\frac{k\lambda }{D\cos\theta }+C_{\varepsilon }\tan\theta .$$

The remnant strain component is represented by C_ε_. Equation (4) has a straight-line form; the plot intersection to the origin gives the crystallite size D, and the slope of the line gives the strain component C_ε_.

Both methods, Scherrer and Williamson–Hall, were applied to the bracelets XRD pattern, and the obtained values are centralized in Table 1.

Scherrer equation results in very small nano-crystallites of only a few nanometers which are not likely to be found in the alloy structure and represent a misquantification of the strains induced at the crystal phase’s level. Therefore, the Williamson–Hall method provides more reliable values, as shown in Table 1, having a great chance to be observed by advanced microstructural investigation. However, these strains induced by the intense cold working cannot be relaxed in a sustainable manner unless the annealing treatment is applied.

**Figure 3:**
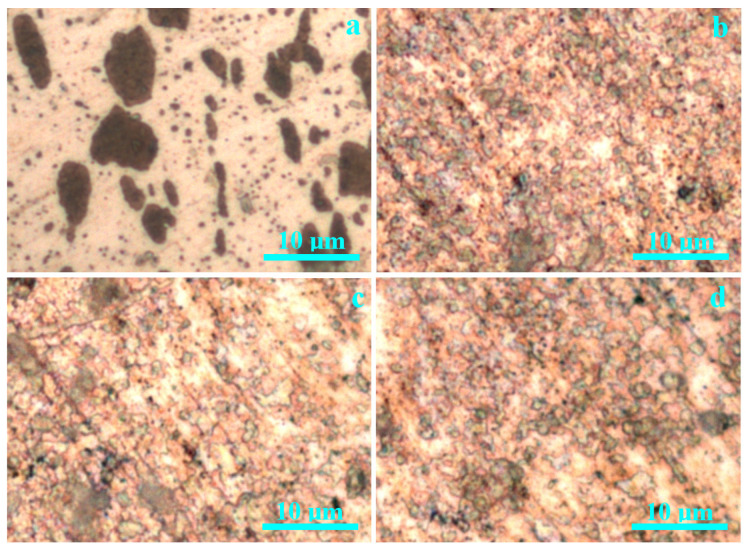
Metallographic images of the investigated pieces volume: (**a**) Dyrrhachium drachma, (**b**) bracelet 1, (**c**) bracelet 2 and (**d**) bracelet 3.

The metallographic investigation, shown in Figure 3, provides more insight into the phase’s distribution within the investigated artifacts. Dyrrhachium drachma has a typical cast microstructure with a large amount of α phase organized in polyhedral grains of about 8 µm in diameter with borders less visible due to the silver matrix coherence and to the reduced etching of the used reagent. The eutectic grains corresponding to the equilibrium α + β phase (e.g., organized in a very refined lamellar structure alternating Ag with Cu sheets [23,24]) have rounded shape and are uniformly distributed in α matrix having brown color due to the ferric chloride etching copper components of the microstructure. Their diameter is situated preponderantly on 8–10 µm, but some smaller eutectic grains are observed around 2.5–3 µm. Small inclusions are observed having grey–black nuance under chemical etch and most likely belong to the sand grains from the casting mold.

The dies struck applied on the cast round pellet has a minor influence on the microstructure, causing only a small elongation of some eutectic grains due to the suddenly compressive effort induced by the hammering.

All bracelet samples have similar microstructures revealed by the metallographic investigation in Figure 3b–d. White spots correspond to α grains; eutectic grains appear in orange hue due to the refined intercalation of α with β sheets. The grain structure is much kneaded with the eutectic grains, severely destructured and scrambled into the α matrix. Therefore, α grains are considerably divided into small fractions difficult to be quantified by their diameter because of the irregular shape but still allow observing that the sizes are situated below 1 µm. Several dark spots appear associated with a grey hue, indicating the significant presence of nonmetallic inclusions.

The dark aspect indicates the presence of amorphous carbon or traces of iron from the metallurgic process like the literature data concerning ancient artifacts [25]. Such an appearance would have been found mainly on the bracelet surface, not in the polished metallographic samples. Moreover, their dissolution within adjacent grains like grey hue indicates an intensive intermixing of the microstructural constituents. Generally, the cold working of alloy slabs induces the grains texturing under the drawing of rolling direction [26,27]. The nonmetallic inclusions and the lack of specific texturing within the bracelets indicates that they were included into the alloy bulk during working procedures, a fact which requires detailed discussion regarding a close correlation with the morphological aspects.

### 3.2. Sample’s Morphology and Nanostructure

**Figure 4:**
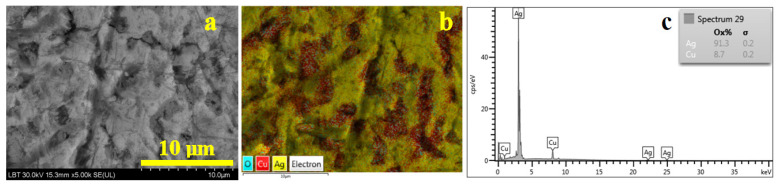
SEM investigation of the Dyrrhachium drachma surface: (**a**) SEI image revealing the morphological aspects, (**b**) BSE image with the elemental distribution map and (**c**) EDS spectrum.

Dyrrhachium drachma surface has a worn surface that affects the grain structure, shown in Figure 4a, with partly scratched α grains maintaining their cohesion. The worn effect erodes the eutectic grains which appear slightly eroded, forming local topographic depressions.

The elemental map in Figure 4b clearly reveals the α matrix (yellow nuance) containing rounded eutectic grains (red coloration) in good agreement with the metallographic microscopy. The turquoise label belongs to oxygen which is less observed on the surface, bringing greenish hues to the deeper scratches having mild oxidation (below the XRD detection limit). Therefore, the elemental composition of the alloy was determined by subtracting the oxygen component within the spectrum in Figure 4c. It results in a silver content of 91.3 wt.% Ag and 8.7 wt.% Cu. It certainly corresponds to the hypoeutectic region of the Ag–Cu phase diagram [28]. Overall, the cast characteristics of the drachma clearly appear on its surface microstructure, having significant influence on the worn characteristics. Also, it indicates that the coin was in circulation for a relatively long time.

**Figure 5:**
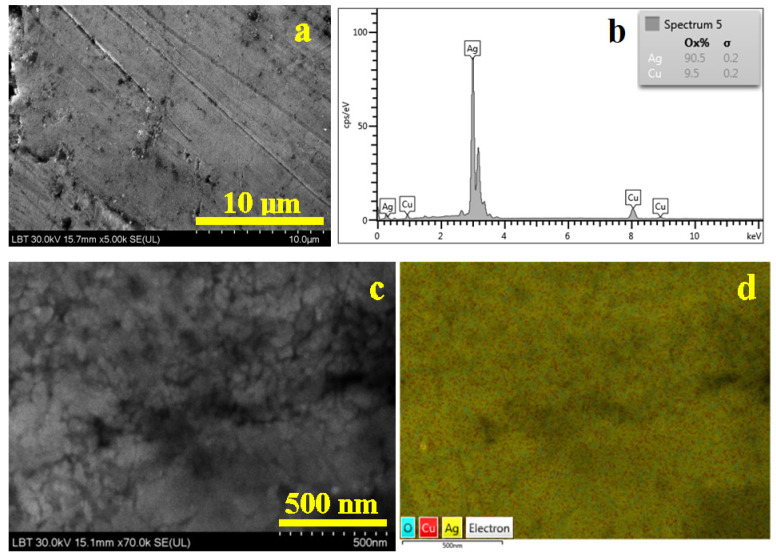
SEM image of the bracelet 1 surface: (**a**) SEI image revealing the morphological aspects, (**b**) EDS spectrum, (**c**) nanostructural detail and (**d**) BSE image with the elemental distribution map for the nanostructural detail.

The surface of bracelet 1 has a totally changed morphology, as shown in Figure 5a. It has worn marks represented by several scratches randomly disposed on the image’s observation field. There are also some small depressions with a darkened aspect which correlates with the nonmetallic inclusions observed at the metallographic observation. These nonmetallic inclusions keep their equiaxed aspect but with irregular sides sustaining their dispersion through intensive cold working of the metal piece. Alloy grains cannot be distinguished at this magnification. However, the alloy composition was measured by the EDS spectrum in Figure 5b in a region free of nonmetallic inclusions. The bracelet’s surface has a mild oxidation below the XRD detection limit but sufficient to be detected by SEM–EDX, and thus oxygen was subtracted from the counting.

The bracelet 1 surface was observed at the same magnification as Dyrrhachium drachma. Therefore, we proceed with inspection of the bracelet’s surface in high-magnification operation mode at ×70,000 (a scale bar of 500 nm), as shown in Figure 5c. The nanostructural detail was taken in a smooth area free of scratches and nonmetallic inclusions. It reveals a dense structure of nanograins having irregular shapes caused by the advanced destructuring of the initial cast α phase and eutectic grains. Overall, assuming an equiaxed shape of the nano grain, their size results around 55 nm. The elemental map in Figure 5 indicates an advanced mixing of the eutectic grains cannot be detected from the α phase; in fact, copper spots appear uniformly distributed within silver mass. It confirms the metallographic observations.

**Figure 6:**
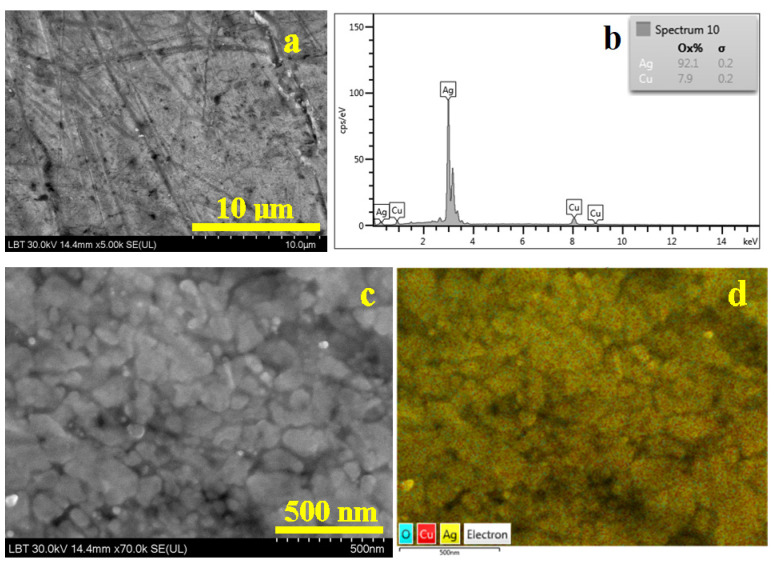
SEM image of the bracelet 2 surface: (**a**) SEI image revealing the morphological aspects, (**b**) EDS spectrum, (**c**) nanostructural detail and (**d**) BSE image with the elemental distribution map for the nanostructural detail.

Bracelet 2 has more randomly dispersed worn scratches on its surface but less nonmetallic inclusions, as shown in Figure 6a. It makes it easy to choose the proper area for the EDS spectrum in Figure 6b by revealing the alloy compound. The surface also has minor oxidation which must be subtracted from the elemental analysis counting.

The nanostructural details in Figure 6c reveal a more heterogeneous arrangement of the nanograins, with most of them having boulder-like shape and sizes preponderantly about 95 nm, but very often they are surrounded by some finer ones indicating advanced intra-granular fragmenting. It would indicate that the eutectic grains were more intensively fragmented than α grains. This assumption is infirmed by the elemental map in Figure 6d revealing a uniform distribution of copper in the silver mass.

**Figure 7:**
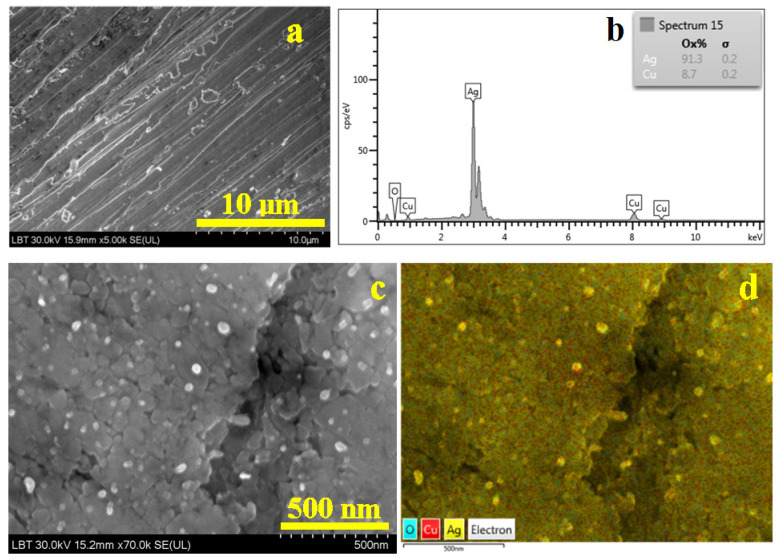
SEM image of the bracelet 3 surface: (**a**) SEI image revealing the morphological aspects, (**b**) EDS spectrum, (**c**) nanostructural detail and (**d**) BSE image with the elemental distribution map for the nanostructural detail.

The surface of bracelet 3 reveals parallel flow marks indicating the alloy texturing during the cold working. The edges of the flow marks have worn marks making them brighter in SEI image. The nonmetallic inclusions are scarcer; few of them can be observed in the upper left corner in Figure 7a.

EDS spectrum in Figure 7b reveals the alloy composition taking into consideration the mild oxidation of the surface. The nanostructural detail in Figure 7c reveals a cold deformation flow mark looking like a depression positioned vertically in the left side of the image. It looks like two layers of the same nanostructured material were pressed one to another until they became the same bulk. The nanograins also have fragmented boulder-like shapes and sizes of about 75 nm. It is interesting that the nanostructure has embedded some white rounded nanograins into the material bulk. The elemental map in Figure 7d allows us to observe that these rounded grains are in fact pure α phase; the yellow nuance clearly shows that they are free of mild oxidation. The rest of the bulk mass has a uniform distribution of copper into silver mass.

SEM observations are centralized in Table 2, revealing that the overall composition of the alloy from the coin is found in all bracelets with minor variations. The grain size decreases about 100 times from the coin’s microstructure to the bracelet’s nanostructure. It is a severe structural modification induced by severe deformation.

**Figure 8:**
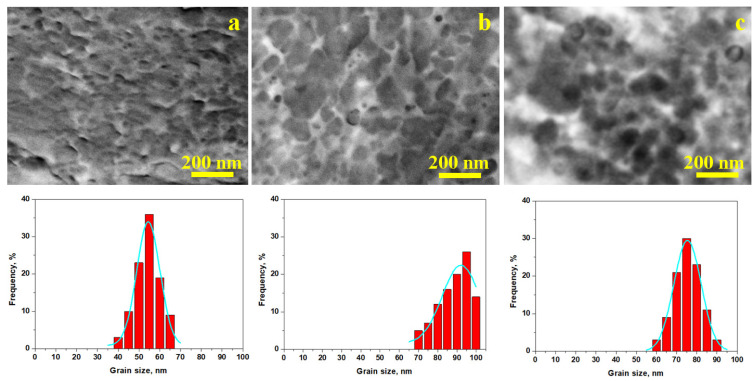
TEM image of the bracelet samples: (**a**) bracelet 1, (**b**) bracelet 2 and (**c**) bracelet 3. The grain size distribution plots are presented below each TEM image. The blue line in the grain size distribution histograms represent the Gaussian fitting curve.

TEM investigation of solid bulk materials is impossible because the accelerated electron beam cannot penetrate the samples depth. Therefore, a thin film of collodion was applied on the bracelet samples in the area observed by SEM. Collodion film solidification ensure a thin replica of the surface microstructure, allowing its observation under TEM conditions obtaining the images in Figure 8.

TEM images in Figure 8 allow better observing of the grain’s nanostructure insight. Ferric chloride reagent used for metallographic investigation etches only copper, bringing dark aspects to the eutectic grains and keeping α phase grains un-etched. Thus, the eutectic distribution within α phase grains borders is evidenced by bright contrast as a consequence of the chemical etching process, while the silver grains appear darker. The uniform distribution of eutectic grains regarding α phase grains gives a valuable clue indicating that it was partly melted during the silver bulk consolidation, suggesting that the coins were transformed into bracelet bars by pressure welding. It most likely happened closely to the eutectic temperature of 779 °C. On the other hand, α phase grains have irregular margins as a consequence of the intensive cold working associated with fine parallel lines inside the grain, indicating their random orientation. Unfortunately, the collodion replica does not allow their observation at high resolution. SEM–EBSD investigation is a powerful tool able to elucidate the crystallographic orientation of grains, but unfortunately it is unavailable at the current stage of investigation. Thus, HR-TEM associated with SEM-EBSD would be the next step of advancement of these precious artifacts for Romanian Cultural Heritage.

Nanostructural differences were also evidenced in TEM images, indicating an advanced grain refinement with a narrow distribution of the grain sizes in bracelet 1, as shown in Figure 8a, and the Gaussian fitting confirmed the SEM observation. The precise mean value with the corresponding standard deviation is presented in Table 2. Bracelet 2, shown in Figure 8b, has a broader grain size distribution, but the average value is given by the dominant size of 95 nm in good observation with SEM measurements. The histogram distribution in Figure 8c also has a narrow aspect with dominant size of about 75 nm, the mean value and corresponding standard deviation being presented in Table 2.

### 3.3. Mechanical Properties

**Figure 9:**
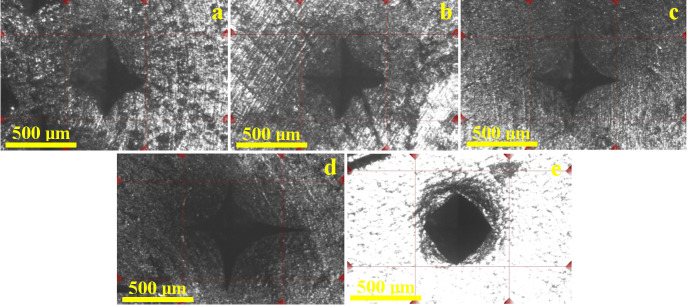
Reflected light microscopy images of the Vickers microhardness indentations: (**a**) bracelet 1, (**b**) bracelet 2, (**c**) bracelet 3, (**d**) Dyrrhachium drachma and (**e**) Annealed silver ingot 999.9‰.

Unfortunately, the bracelets cannot be subjected to tensile testing for investigating their mechanical properties, but microhardness testing allows a proper quantitative observation of their mechanical behavior, as shown in Figure 9.

Such cold-hardened nanostructure definitely would resist better to the penetration of the Vickers indenter compared to the cast microstructure of the Dyrrhachium drachma. Still, a comparative standard must be investigated. Therefore, a pure 999.9 silver ingot in annealed state was used for this purpose. The aspect of indentation marks reveals slim cross marks for all bracelets, as shown in Figure 9a–c, while the coin’s indentation has a more deeply rhombic shape and a bigger size, as shown in Figure 9d. The annealed ingot has a greater plasticity than the indentation cross and is less visible, while the rhombic shape of the edges becomes more visible, as shown in Figure 9e.

**Figure 10:**
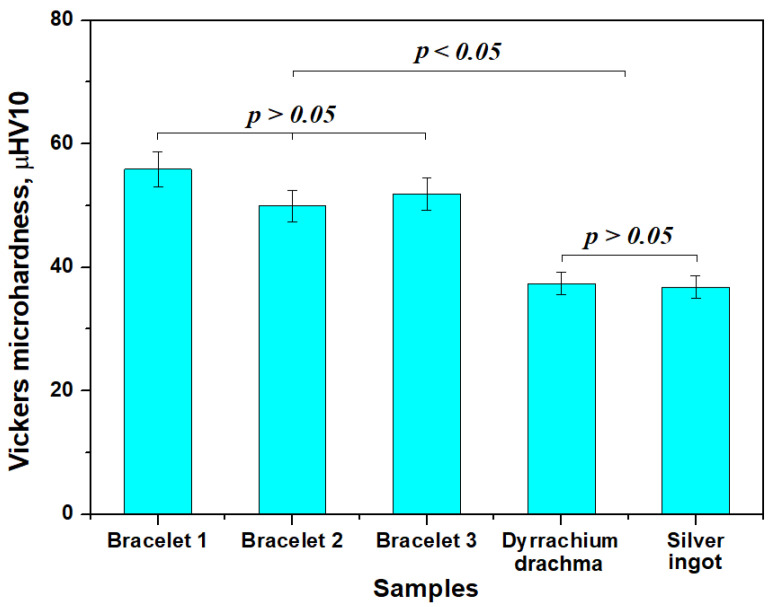
Mean values of Vickers microhardness variation.

The mean values of microhardness resulted for the investigated bracelets ranges from 50 to 56 µHV10 in the samples, as shown in Figure 10. Bracelet 1 is the tougher one because of its smaller nanostructure, while bracelet 2 is the softer one because of the bigger nanograins. However, the microhardness resulted for all bracelets forms a statistically relevant group (*p* > 0.05). On the other hand, the Dyrrachium drachma has a lower microhardness comparable with the annealed pure silver ingot, being situated at about 37 HV10 and forming the second statistically relevant group. The statistical analysis also reveals a significant difference between the two identified groups (*p* < 0.05). Thus, the microhardness investigation confirms the assumption regarding the cold hardening and its subsequent nanostructure development.

## 4. Discussion

Dyrrhachium drachmas were very popular coins within antiquity because of their stable weight of 2.98 g (diameter about 18 mm having side approximate round) and a silver title about 90–95 wt.% depending on the literature source and their archeological deposits [13,29]. These drachmas were issued during the Roman protectorate on the ancient Dyrrhachium during 229–30 BC, and their quality was controlled and ensured by two magistrates, their names being inscribed on the obverse and reverse, in our case Damenos on the obverse and Meniskos on the reverse. The magistrate names combination and particular style of the coins allow a proper date of their issue during the circulation period [30]. The good quality of Dyrrhachium drachmas make them want for commercial exchange into Dacian territories being related to the salt and slave commerce as well as stipendium for the mercenary activities [31].

As observed, the historical context explains the massive presence of such drachmas in the Cehei hoard. The higher amount of good silver coins sustains the archeologist’s arguments that they were used as raw material for jewelry production. The initial assumption made by the archeologists in 1986 indicates their re-melting and casting in ingots which were further processed by hammering. It is a reasonable assumption which is partly unsustained by the current results. The coins re-melting would collect the nonmetallic impurities and oxides into the slag, conducting to a cleaner cast microstructure [32,33]. It would result in a better microstructural distribution than the one observed in drachma because of advanced removal of mineral inclusions embedded from the original casting mold. The successive hammering (there is no archeological evidence for metal rolling in Dacian workshops) would have textured the grain shapes under the metal flow direction [34], a fact not sustained by our observations. The intermediary re-crystallization annealing would restore the equiaxed shape of the grains, inducing a regulated microstructure regarding α and eutectic grains in a similar manner with the data reported in the literature [35].

The bracelets’ kneaded microstructure does not resemble any conventional technological pathways available nowadays. There is a forgotten welding technique by hammering the welded parts when they are heated at high temperature but below the melting point, ensuring the alloy diffusion from one part into another into a continuous microstructure. This technique was used by blacksmiths for welding the chain links known as pressure welding [36,37]. Thus, we proposed another technological method for the conversion of Dyrrhachium drachmas into bracelets bars without re-melting. The coin pile is heated at high temperature below the melting point in a range beginning closely below eutectic temperature of 779 °C and closing slightly above this temperature. It allows partial melting of the eutectic grains while α grains remain in solid state but have an increased plasticity. Subsequently, hammering of the heated coin pile causes their welding into a single metal piece which is continuously modeled into a bar. This procedure ensures the kneading of α grains while partly melted eutectic ensures their cohesion, a fact sustained by the TEM images in Figure 8. Heating in coal-auctioned furnaces and hammering on the iron anvil induces significantly the observed nonmetallic inclusions. The proposed technological pathway is fully sustained by the metallographic analysis results.

The further cold hammering of the bracelets activates the cold hardening mechanism, causing an extra refinement of the microstructure, turning its grains into nanostructured ones. The nano-crystallites induce diffraction peaks broadening due to their size reduction and because of the remnant strains at the crystal lattice level, and thus the Williamson–Hall method allows a proper quantification of the nanograins size [21,22], values confirmed by the high magnification SEM images. Thus, the nanograins’ size is about 55 nm for bracelet 1, 95 nm for bracelet 2 and 75 nm for bracelet 3. They have preponderantly boulder-like shapes with irregular margins well embedded within the eutectic grains looking diffused within the α matrix. The elemental maps clearly indicate uniform distribution of Cu within silver mass without contouring precise eutectic grains. This nanostructural refinement indicates that the silver alloy bar was produced by pressure welding instead of re-melting followed by casting.

It is well known that silver melting has significant loss through evaporation if a proper artificial slag layer is induced during crucible heating [38,39]; thus, the ancient re-melting would lead to a significant loss in the silver amount. Applying the pressure welding method has the advantage of keeping the silver amount about the same, ensuring a maximization of the coins’ conversion to jewelry. Drachmas processing through melting as Chirila and Matei stated in 1986 [14] would have caused a sensitive decrease in the silver title because of its peculiar evaporation in a melted state at high temperatures. Our SEM observation reveals that the drachma’s silver title is similar with the silver title of the bracelets. Thus, our results sustain that the drachmas were processed into raw bars for bracelets through pressure welding techniques which avoid silver evaporation through the melted bath.

Nanostructuring through cold hardening of the silver alloy induces a significant increase in the Vickers microhardness of the bracelets in comparison with the coin having similar microhardness with the annealed pure silver ingot, confirming once more that the proposed technology was applied to obtain the bracelets. The bracelets’ increased values of the Vickers microhardness clearly sustain their nano-structuring compared to Dyrrhachium drachma and annealed silver ingot. The literature reveals that the coin’s surface is very affected by the strong indentations like the demonetization marks imprinted in the modern issues [40,41]. Thus, smaller and thin indentations on the bracelets contrast with the bigger and wider indentations of the coin’s surface.

The current study’s limitations reside in nondestructive methods which are absolutely necessary when valuable archeological artifacts are involved. Tensile strength testing and subsequent SEM fractography would have brought additional information of the production technology, but they are not available for now. The future archeological finds maybe will bring out some similar bracelets fragments which perhaps allow tensile testing. A further nondestructive investigation should be conducted using Electron Backscatter Diffraction (EBSD) for evidencing the crystallographic orientation of the grains within the investigated alloy samples [42,43] and to find similar archeological artifacts to verify if the proposed technology is confirmed in other similar artifacts. The validation of the proposed manufacturing technology should be proved in further research by replication of the process with materials as close as possible to the original ones and their comparison with the other metallurgical archeological finds in Salaj County.

## 5. Conclusions

The high tenacity of almost pure silver bracelets from the Cehei Dacian hoard is caused by the advanced refinement of the microstructure induced by the cold hardening process occurring during cold working of the final steps. XRD and SEM observation reveal that all three bracelets are in fact nanostructured with nanograins of 55 nm for bracelet 1, 95 nm for bracelet 2 and 75 nm for bracelet 3. The bracelet alloy nano-structuring causes its microhardness to increase to 56 μHV10 for bracelet 1; 50 μHV10 for bracelet 2 and 52 μHV10 for bracelet 3, while the Dyrrhachium drachma and annealed pure silver ingot hardness is situated around 37 HV10. Finally, it results that the coins were used as raw material for the bracelets, but it was processed by pressure welding instead of casting preserving the metal amount. The advanced cold working during the final step of bracelets manufacturing ensures the grains nanostructuring.

## Figures and Tables

**Table 1:** The data calculated on the XRD patterns.

Sample	Ag, wt.%	Crystallite Size, nm	
		Scherrer	Williamson–Hall
Bracelet 1	91	5.22	50
Bracelet 2	90	17.46	80
Bracelet 3	95	8.49	70

**Table 2:** The data resulted from SEM investigation.

Sample	Ag, wt.%	Cu, wt.%	Grain Size, nm	
			SEM	TEM
Drachma	91.3	8.7	8000	-
Bracelet 1	90.5	9.5	55	55 ± 15
Bracelet 2	92.1	7.9	95	95 ± 25
Bracelet 3	91.3	8.7	75	75 ± 20

## Data Availability

The original contributions presented in the study are included in the article.

## Abbreviations

The following abbreviations are used in this manuscript:

BSE	Backscatter Electron Image
EBSD	Electron Backscatter Diffraction
EDS	Energy Dispersive Spectroscopy
RIR	Relative Intensity Ratio
SEM	Scanning Electron Microscopy
SEI	Secondary Electron Image
TEM	Transmission Electron Microscopy
XRD	X-ray Diffraction
